# Rural–urban health disparities among older adults in South Africa

**DOI:** 10.4102/phcfm.v11i1.1890

**Published:** 2019-06-19

**Authors:** Karl Peltzer, Nancy Phaswana-Mafuya, Supa Pengpid

**Affiliations:** 1 Department of Research and Innovation, North West University, Potchefstroom, South Africa; 2 HIV/AIDS/STIs and TB (HAST) Research Programme, Human Sciences Research Council, Pretoria, South Africa; 3 ASEAN Institute for Health Development, Mahidol University, Nakhonpathom, Thailand

**Keywords:** rural–urban, health status, chronic conditions, disparities, older adults, South Africa

## Abstract

**Background:**

There are limited studies assessing rural–urban disparities among older adults in Africa including South Africa.

**Aim:**

This study explores rural–urban health disparities among older adults in a population-based survey in South Africa.

**Setting:**

Data for this study emanated from the 2008 study on ‘Global Ageing and Adult Health (SAGE) wave 1’ (*N* = 3280) aged 50 years or older in South Africa.

**Methods:**

Associations between exposure variables and outcome variables (health status variables and chronic conditions) were examined through bivariate analyses and multivariable logistic regression.

**Results:**

Rural dwellers were more likely to be older, black African and had lower education and wealth than urban dwellers. Rural and urban dwellers reported a similar prevalence of self-rated health status, quality of life, severe functional disability, arthritis, asthma, lung disease, hypertension, obesity, underweight, stroke and/or angina, low vision, depression, anxiety and nocturnal sleep problems. Adjusting for socio-demographic and health risk behaviour variables, urban dwellers had a higher prevalence of diabetes (OR: 2.36, 95% CI: 1.37, 4.04), edentulism (OR: 2.79, 95% CI: 1.27, 6.09) and cognitive functioning (OR: 1.91, 95% CI: 1.27, 2.85) than rural dwellers.

**Conclusion:**

There are some rural–urban health disparities in South Africa, that is, urban dwellers had a higher prevalence of diabetes, edentulism and cognitive functioning than rural ones. Understanding these rural–urban health variations may help in developing better strategies to improve health across geolocality in South Africa.

## Introduction

Noncommunicable diseases (NCDs) have become one of the world’s biggest public health problems, contributing to premature death, disability, productivity losses and high health care costs that may eventually affect the achievement of the sustainable development goals.^1^ Ageing is often associated with decline in health status characterised by limited physical functioning, increase in chronic diseases as well as decrease in cognitive functioning.^2,3^ Social determinants of NCDs include socio-economic factors (e.g. poverty, inequality, rural–urban differences)^4^ and risky health behavioural repertoire that may have developed in earlier years (e.g. such as smoking, alcohol abuse, limited physical activity and unhealthy diet with high cholesterol, salt and saturated fats lacking fresh fruits and vegetables).^2,3,4^

In many parts of the world, rural–urban health disparities persist in terms of socio-demographics, health care access, health status and prevalence of chronic conditions. Concerning socio-demographics, rural subjects were significantly younger, more likely to be women, widowed as well as have lower education and income than their urban counterparts in China.^5^ Similarly, older rural adults had significantly lower educational levels and less adequate income than older urban ones.^6^ Compared to single individuals, widowed middle and older aged South Africans living in urban communities were more likely to have hypertension and diabetes.^7^ Urban residence was found to be associated with better access to health care (hospital admission, private transport to a health facility, shorter travel distance to a health facility, private outpatient care, less often experienced catastrophic health care costs and utilisation of higher level public facilities) in South Africa.^8^

Regarding health status, rural older dwellers, including in South Africa, reported significantly lower overall health status and lower quality of life than urban ones.^5,6,9^ Among older adults in China, urban and urban-to-urban residents had the highest level of cognitive function, while rural and rural-to-rural residents had the poorest cognitive function.^10^ In Japan, the prevalence of cognitive impairment was higher in rural than urban areas.^11^ Rural residence in China was found to have higher functional limitation than urban residence,^12^ while in Bangladesh, urban dwellers reported more difficulties with activities of daily living or functional disability than rural dwellers.^13^ The prevalence of sarcopenia was significantly higher in rural elders than in urban elders in China.^14^

Among older adults, urban residence has been found to be associated with a higher prevalence of asthma morbidity,^15^ chronic lung disease,^16^ stroke,^17^ angina,^16^ hypertension^16,17^ and diabetes.^7,15,16,18^ Several studies found a higher prevalence of underweight or malnutrished among rural people rather than urban older adults.^6,19^ Being overweight or obese was found to be higher among urban than rural residents in Iran^19^ and South Africa,^7^ while in the USA, obesity was markedly higher among adults from rural versus urban areas.^20^ As a marker of oral health, edentulism was more prevalent in urban than rural older adults in Ghana.^21^

In terms of mental health, urban residence was associated with a higher prevalence of depression in China,^16^ while in other studies in China, rural subjects had a significantly higher proportion of depressive symptomatology than urban dwellers.^5,22^ Regarding health risk behaviours for NCDs, for example, the prevalence of alcohol use was significantly higher in the urban than rural areas in South Africa,^7,18^ vigorous physical activity was more prevalent among rural than urban dwellers in South Africa^23^ and tobacco use was significantly higher in the rural than urban areas in India.^24^ Compared to their rural counterparts, urban youth in South Africa were more likely to smoke and be physically inactive.^25^

There is a dearth of studies examining rural–urban differences in health at older ages on the African continent, including South Africa.^18^ This study examines rural–urban differences among older South Africans who participated in the Study of Global Ageing and Adults Health (SAGE). This study provides critical evidence for health policy planning for South Africa, the fastest ageing country in the African continent.

This study provides a general picture of rural–urban health disparities in relation to health status and chronic conditions.

## Research methods and design

### Sample and procedure

This article utilised cross-sectional data from SAGE, a population-based study that had a sample of 3840 older South Africans aged 50+ years. A two-stage probability sample was used and it produced representative estimates nationally and provincially by geographic type (urban and rural) and population group (black, mixed race, Indian or Asian and white); more details are discussed in Kowal et al.^26^ A good response rate (77%) was obtained.

### Measurements and tools

*Socio-demographic* variables assessed were: age, gender, educational level in years, geolocation, population or racial group and economic or wealth status.

The other complete measurements have been described previously,^27^ and are therefore only listed here*: Economic or wealth status in* wealth quintiles, *Health risk behaviours* (daily tobacco use, problem drinking) (≥ 10 drinks or week), physical inactivity (< 600 metabolic equivalent minutes per week) and inadequate fruit and vegetable consumption (< 5 servings a day). *Health status variables:* poor self-reported health status (bad or very bad general health), weak grip strength (< 30 kg for men and < 20 kg for women), functional disability (according to the International Classification of Functioning, Disability and Health, 50% – 100% were defined as severe or extreme functional disability, using the 12-item WHO Disability Assessment Schedule, version 2 [WHODAS-II], high cognitive functioning [a median score of 48 or more]) and quality of life (assessed with the eight-item ‘World Health Organization Quality of Life’ scale). *Chronic conditions:* arthritis (symptoms algorithm based); asthma (self-reported diagnosed and/or symptoms algorithm based); lung disease and depression (symptoms algorithm based according to the International Classification of Diseases’ 10th revision diagnostic criteria for research for depressive episodes); obesity (standard height and weight measures, 30 kg/m^2^); diabetes, stroke, angina and edentulism (self-reported diagnoses); anxiety, sleeping problems (self-reported); measured hypertension (based on three averaged blood pressure measurements and/or taking antihypertensive medication) and measured low vision using a tumbling ‘E’ LogMAR chart.^27^

### Data analysis

STATA software version 13.0 (Stata Corporation, College Station, TX, United States [US]) was used to analyse data taking the sampling design into consideration. Differences in proportions and means of exposure variables were tested using Chi-square and *t*-tests. Odds ratios (OR) were used to determine associations between exposure (socio-demographics and health risk behaviours) and outcome (health status and chronic conditions)variables using multivariate logistic regression. Weighted percentages have been presented in the tables. The *p*-value (< 5%) and 95% confidence intervals were used to indicate statistical significance taking the complex sample design into account.

### Ethical considerations

All essential study approvals were secured, that is, the Human Sciences Research Council (HSRC), Research Ethics Committee (Protocol REC 5/13/04/06) and the National Department of Health. Participants also provided voluntary written informed consent prior to participating in the study.

## Results

### Sample characeristics

The study sample had 3280 individuals aged 50 years or older (44.1% men and 55.9% women). The sample consisted of black Africans (74.0%), followed by mixed race (12.8%), white people (9.3%) and Indian or Asian people (3.8%). Rural dwellers were a little older and had a higher proportion of black Africans than urban dwellers. Urban dwellers had a higher formal education and were wealthier than rural dwellers. However, no rural–urban differences were observed in terms of sex, marital status, tobacco use, problem drinking, physical inactivity and inadequate fruit and vegetable consumption (see Table 1).

**TABLE 1:** Exposure variables by rural–urban residence (*N* = 3840).

Variable	Rural†				Urban‡				*p*
	*M*	SD	*n*	%	*M*	SD	*n*	%	
**Age**	63.6	10.2	-	-	62.3	9.4	-	-	< 0.001
**Sex**									
Female	-	-	715	57.1	-	-	1485	55.2	0.404
Male	-	-	561	42.9	-	-	1076	44.8	
**Population group**	-	-			-				
Black African	-	-	920	90.8	-	-	1131	64.8	0.003
White African	-	-	51	4.0	-	-	218	12.2	
Mixed race	-	-	110	3.8	-	-	545	17.8	
Indian or Asian African	-	-	22	1.5	-	-	285	5.1	
**Education**									
< 7 years	-	-	785	70.7	-	-	901	41.3	< 0.001
8–11 years	-	-	205	21.5	-	-	846	38.2	
12 years or more	-	-	76	7.7	-	-	339	20.5	
**Wealth**	-	-							
Low	-	-	722	58.8	-	-	-	30.7	< 0.001
Medium	-	-	250	19.7	-	-	-	17.4	
High	-	-	299	21.5	-	-	-	51.8	
**Marital status**									
Not married, single, widowed	-	-	129	13.0	-	-	383	15.0	0.666
Married, cohabiting	-	-	1115	87.0	-	-	2139	85.0	
**Daily tobacco use**	-	-	285	21.7		-	523	19.7	0.602
**Problem drinking**	-	-	47	3.1	-	-	111	4.1	0.414
**Physical inactivity**	-	-	780	58.1	-	-	1673	61.8	0.674
**Inadequate fruit and vegetable consumption**	-	-	1048	75.1	-	-	1782	64.9	0.268

† , *n* = 1276.

‡ , *n* = 2561.

### Health status and chronic conditions

Table 2 describes health status, physical chronic and poor mental health conditions by rural–urban residence (see Table 2). In terms of health status, 22.3% rural older adults and 14.9% urban older adults reported poor self-rated health status. Regarding physical chronic conditions, 5.6% rural and 11.1% urban older adults self-reported the diagnosis of diabetes. As regards to mental health, 3.7% of rural and 2.5% of urban older adults were classified as having major depression.

**TABLE 2:** Prevalence of health status and chronic conditions by rural–urban residence.

Variable	Rural		Urban	
	*n*	%	*n*	%
**Health status**				
Poor self-rated health	230	22.3	386	14.9
Quality of life (low)	385	36.2	571	24.3
Grip strength (weak)	242	28.0	537	28.4
Functional disability (severe)	142	14.6	223	9.2
Cognitive functioning (high)	471	38.9	1322	58.9
**Physical chronic conditions**				
Arthritis	294	25.9	757	29.8
Asthma	115	12.1	254	11.7
Lung disease	86	6.4	153	6.4
Hypertension	916	77.5	1923	77.2
Underweight	65	5.8	120	4.1
Obesity	445	42.5	1118	49.3
Diabetes	62	5.6	297	11.1
Stroke and/or angina	93	7.6	245	9.1
Edentulism	44	3.5	324	11.1
Low vision	502	43.4	1060	43.1
**Mental health**				
Depression	38	3.7	80	2.5
Anxiety	119	11.5	171	8.5
Nocturnal sleep problem	123	10.8	205	8.5

Adjusting for socio-demographic factors and health risk behaviour variables, urban dwellers had a higher prevalence of diabetes (OR: 2.36, CI: 1.37, 4.04), edentulism (OR: 2.79, CI: 1.27, 6.09) and cognitive functioning (OR: 1.91, CI: 1.27, 2.85) than rural dwellers. No significant rural–urban differences were found on the other three health status measures (self-rated health status, quality of life and severe functional disability) and other chronic conditions (arthritis, asthma, lung disease, hypertension, obesity, underweight, stroke and/or angina, low vision, depression, anxiety and nocturnal sleep problems) (see Table 3).

**TABLE 3:** Odds ratios for health status and chronic conditions by rural–urban residence.

Variable	Rural†	Urban‡		*p*
		OR	95% CI	
**Health status**				
Poor self-rated health				
Unadjusted	1 (Reference)	0.61	0.26, 1.46	0.260
Adjusted§	1 (Reference)	0.77	0.38, 1.55	0.453
Quality of life (low)				
Unadjusted	1 (Reference)	0.57	0.31, 1.05	0.068
Adjusted§	1 (Reference)	0.78	0.44, 1.39	0.389
Grip strength (weak)				
Unadjusted	1 (Reference)	1.02	0.62, 1.69	0.939
Adjusted§	1 (Reference)	1.05	0.65, 1.71	0.831
Functional disability (severe)				
Unadjusted	1 (Reference)	0.60	0.21, 1.69	0.322
Adjusted§	1 (Reference)	0.65	0.23, 1.89	0.423
Cognitive functioning (high)				
Unadjusted	1 (Reference)	2.25	1.40, 3.63	< 0.001
Adjusted§	1 (Reference)	1.91	1.27, 2.85	0.002
**Chronic conditions**				
Arthritis				
Unadjusted	1 (Reference)	1.22	0.78, 1.90	0.381
Adjusted§	1 (Reference)	1.22	0.83, 1.80	0.302
Asthma				
Unadjusted	1 (Reference)	0.96	0.62, 1.49	0.865
Adjusted§	1 (Reference)	0.97	0.70, 1.44	0.838
Lung disease				
Unadjusted	1 (Reference)	1.00	0.51, 1.95	0.998
Adjusted§	1 (Reference)	1.01	0.56, 1.82	0.962
Hypertension				
Unadjusted	1 (Reference)	0.98	0.59, 1.64	0.952
Adjusted§	1 (Reference)	1.00	0.67, 1.49	0.995
Obesity				
Unadjusted	1 (Reference)	1.31	0.83, 2.09	0.240
Adjusted§	1 (Reference)	1.25	0.77, 2.01	0.353
Underweight				
Unadjusted	1 (Reference)	0.82	0.49, 1.37	0.437
Adjusted§	1 (Reference)	0.79	0.37, 1.69	0.537
Diabetes				
Unadjusted	1 (Reference)	2.11	1.48, 3.01	< 0.001
Adjusted§	1 (Reference)	2.36	1.37, 4.04	0.003
Stroke and/or angina				
Unadjusted	1 (Reference)	1.21	0.82, 1.77	0.324
Adjusted§	1 (Reference)	0.94	0.63, 1.40	0.743
Edentulism				
Unadjusted	1 (Reference)	3.43	1.50, 7.87	0.004
Adjusted§	1 (Reference)	2.79	1.27, 6.09	0.012
Low vision				
Unadjusted	1 (Reference)	0.99	0.61, 1.61	0.964
Adjusted§	1 (Reference)	0.86	0.55, 1.35	0.508
Depression				
Unadjusted	1 (Reference)	0.66	0.33, 1.32	0.233
Adjusted§	1 (Reference)	0.73	0.33, 1.64	0.436
Anxiety				
Unadjusted	1 (Reference)	0.73	0.35, 1.55	0.406
Adjusted§	1 (Reference)	0.82	0.45, 1.50	0.516
Nocturnal sleep problems				
Unadjusted	1 (Reference)	0.68	0.32, 1.47	0.317
Adjusted§	1 (Reference)	0.82	0.44, 1.55	0.539

OR, odds ratio; CI, confidence interval.

† , *n* = 2202.

‡ , *n* = 1638.

§ , Adjusted for age, sex, population group, education, wealth, tobacco use, alcohol use, physical inactivity and fruit and vegetable consumption.

## Discussion

This study examined rural–urban health disparities among older adults in a national sample in South Africa. Rural older adults in this study were predominantly black African (90.8%), while urban ones were proportionally more white and mixed race (30%). As found in previous studies in developing countries, such as China,^5,6^ rural older adults in this study had less education and were less wealthy than urban older adults were. This could mean that rural older adults have less access to health care services than urban dwellers, and health care access should be improved for rural older adults in South Africa.^8^

Regarding health status, this study found that poor self-rated health and low quality of life were higher among rural than urban older adults, but this was not significantly higher. Previous studies have found that rural subjects reported significant lower overall health status and lower quality of life than urban dwellers.^5,6,28^ Poorer perceived health status and lower quality of life in rural dwellers may be related to lower socio-economic status and higher unemployment, which, in turn, reduce affordability of good nutrition and access to health care.^9^ This highlights the importance of an integrated national development plan in which the provision of health is not in isolation but situated within a larger developmental context.

Functional disability was in this study also higher among rural than urban older adults, but also not significantly. Previous studies found mixed results regarding the geographic determinants of functional disability.^12,13^ Although previous studies^14^ found a relationship between rural older adults and sarcopenia (or weak hand grip strength), this study did not find any rural–urban differences. Consistent with previous studies,^10,11^ this study found a higher cognitive functioning in urban compared to rural older adults. Better cognition in later life has been associated with higher levels of education.^29,30^ Our urban sample had much more formal education than the rural sample, which may explain the higher levels of cognitive functioning among the urban older adults. It is possible that early and later life transitions of migration to urban areas, seeking and receiving higher levels of education, impacted on the levels of cognitive functioning in South Africa.^10^ Further studies should validate these findings and develop explanatory models that could help in describing the mechanisms responsible for the found associations.

Regarding chronic conditions, urban residence was significantly associated with diabetes and edentulism in this study, as found in previous studies.^15,16,21^ The higher self-reported prevalence of diabetes in urban rather than rural areas may be related to a higher prevalence of combined risk factors, such as dietary changes, physical inactivity and obesity,^16^ and older adults in rural areas may have less access to being diagnosed with diabetes than in urban areas.^16^ Possible reasons for the higher prevalence of edentulism in urban areas may be related to dietary changes such as increased consumption of refined sugars, which may lead to caries and tooth loss,^21^ and dental care services and tooth extractions are more likely to be available in urban than rural areas. The study found that the prevalence of underweight was higher in rural than urban older adults, while the prevalence of obesity was higher among urban than rural older adults, but the differences did not reach significant levels. Previous studies^6,19^ confirm the relationship between rural residence and being underweight or malnutrition among older adults, while mixed results were found on urban–rural differences in relation to obesity.^7,19,20^ The study found no rural–urban associations for arthritis, asthma, lung disease, low vision, stroke and/or angina and hypertension, while previous studies found an association between urban residence and asthma morbidity,^15,16^ chronic lung disease,^16^ stroke,^16^ angina^16^ and hypertension.^16,17^

Previous studies^5,16,22^ found mixed results regarding rural–urban differences and mental health indicators such as depression, while this study did not find significant differences for depression, anxiety and nocturnal sleep problems. Surprisingly, contrary to some previous studies,^7,23,24,25^ no rural–urban differences were found for behavioural risk factors of NCDs (tobacco use, problem drinking, physical inactivity and insufficient fruit and vegetable consumption). This could mean that health risk behaviours, such as tobacco use, problem drinking, physical inactivity and inadequate fruit and vegetable consumption, have penetrated into both urban and rural areas in South Africa. Therefore, health behaviour interventions should target rural and urban dwellers equally.

This study provides evidence that can be used to guide health care service provision in South Africa and elsewhere. The findings of this study (largely do not but sometimes do include rural–urban health disparities) should be contextualised given its limitations. Chronic conditions were subjectively reported, that is, based on self-reports or symptom algorithms. The cross-sectional nature of the design makes it difficult to determine causality. In conclusion, it is clear that few rural–urban variations exist in health status and chronic conditions. Understanding these rural–urban health disparities may help in developing better strategies to improve health in rural and urban areas.
